# Challenging the Stereotypes: Unexpected Features of Sexual Exploitation among Homeless and Street-Involved Boys in Western Canada

**DOI:** 10.3390/ijerph18115898

**Published:** 2021-05-31

**Authors:** Elizabeth M. Saewyc, Sneha Shankar, Lindsay A. Pearce, Annie Smith

**Affiliations:** 1 Stigma and Resilience among Vulnerable Youth Centre, School of Nursing, University of British Columbia, T201-2211 Wesbrook Mall, Vancouver, BC V6T 2B5, Canada; sneha.shankar@mcgill.ca (S.S.); lindsayapearce@gmail.com (L.A.P.); 2 McCreary Centre Society, 3552 E. Hastings St., Vancouver, BC V5K 2A7, Canada; annie@mcs.bc.ca

**Keywords:** sexual exploitation, human trafficking, homelessness, adolescent males

## Abstract

Research about the sexual exploitation of homeless and street-involved boys is limited and often combined with that of girls. As aggregation can distort unique issues among genders which are exploited, this study provides information about the context of exploitation for homeless boys. Boys participated in the anonymous, multi-city British Columbia (BC), Canada Homeless and Street-Involved Youth Health paper surveys of 2006 and 2014. Measures included questions about trading sex for money, shelter, or other consideration; age first exploited; for whom; where they were living when first traded sex; gender of exploiters; and demographics. Analyses, separately for younger/older boys, explored the prevalence, timing of exploitation vs. homelessness, and ANOVAs to evaluate the patterns of the age of first exploitation by the genders of exploiters. Just over one in four boys reported exploitation (n = 132), with a median age of 14–15 in most groups. Most were runaway or homeless before their first exploitation, but 25.5% (2006) and 41% (2014) were living with family. Most boys were exploited by women (78%–85%), with 62%–65% were exclusively exploited by women.

## 1. Introduction

The sexual exploitation of young people is a human rights violation and has significant health consequences for most youth who have been exploited. While boys, girls, and transgender youth are all affected, research has tended to focus on the risks for girls [1] and the existing literature about the sexual exploitation of boys is limited [2,3,4]. 

Sexual exploitation in Canadian law includes exchanging money, drugs, gifts, food, services, shelter, transportation, or other considerations for any type of sexual activity with an individual who is 17 years old or younger. It is based on the definition from the United Nations Convention on the Rights of the Child optional protocol introduced in 2000 (Article 2), which identifies the sale of children, child prostitution and child pornography as specific aspects of exploitation from which children need protection [5] (pp. 248–249):

“(a) Sale of children means any act or transaction whereby a child is transferred by any person or group of persons to another for remuneration or any other consideration; (b) Child prostitution means the use of a child in sexual activities for remuneration or any other form of consideration; (c) Child pornography means any representation, by whatever means, of a child engaged in real or simulated explicit sexual activities or any representation of the sexual parts of a child for primarily sexual purposes.” 

It should be noted that these definitions of sexual exploitation do not require force or coercion of children or adolescents, nor do they limit the definition of exploitation to sex purchased by adults, or sex that has been facilitated by a third party, i.e., a pimp, procurer or trafficker. Adolescents who purchase sexual activity from another young person, or purchasers who provide money, goods or services to adolescents directly in exchange for sex rather than to a procurer, still have sexually exploited that adolescent.

In a systematic review examining sexual exploitation among boys, Mitchell et al., highlighted that the term sexual exploitation is used inconsistently in the literature, which presents a challenge for researchers exploring this topic [2]. Some commonly used synonyms for sexual exploitation include human trafficking or sex trafficking, which can include individuals 18 years or older [2,6]. Sex trade, sex exchange, or sex work are also sometimes used, although these terms tend to obscure the exploitative nature of this form of child sexual abuse and imply a level of agency and consent to the sexual exchange that may not be consistent with the emerging capacity of adolescents. Survival sex, or sex in exchange for food or shelter, can still be considered sexual exploitation if the adolescent providing the sex activity is under 18 years old. Although the legal and health sectors recognize that the sexual exploitation of young people is problematic [6,7], the research is hindered by varying definitions, and by studies that do not report the experiences of those under 18 years separately from those 18 years of age and older. Mitchell and colleagues recommend that research about boys who are sexually exploited needs to disaggregate young men who are 18 and older and boys who are 17 and younger, to allow for a nuanced understanding of the contexts in which boys are sexually exploited [2].

Part of the challenge in addressing sexual exploitation is that stereotypes about this abuse persist in Canada and around the world. For instance, the majority of information in the literature and news media depict youth who are exploited as girls and women, and as heterosexual [6,8,9], and policies typically focus on sexual exploitation among girls [10]. This overlooks the risks of sexual exploitation for boys [2,11]. However, a growing number of population-based and large-scale multiple-site studies have found similar rates between boys and girls who have been sexually exploited. For example, school-based surveys in the Canada, Norway, Sweden, and the United States find that a small proportion of youth report trading sex for money or alcohol and drugs, generally 2%–4%; in these studies, boys are as likely or more likely to report exploitation [12,13,14,15,16,17]. Studies among homeless and street-involved youth identify larger proportions of young people who have been sexually exploited, with estimates ranging from 10% to 38% in studies across Canada and the United States over the past 20 years [18,19,20,21,22]. In these studies, rates are not always reported separately for girls and boys, but when they are, some studies find similar rates, e.g., [21] while others find girls are more likely to report sexual exploitation, e.g., [20]. Studies among homeless youth in other parts of the world report an even higher prevalence, for example, 41% of boys in a study of street-involved youth in Pakistan [23] and 45% in Ghana [24]. 

According to Reid, a key vulnerability for boys relates to sexual orientation and sexual identity, with gay and bisexual boys more likely to be exploited than their heterosexual peers [25]. Other studies find lesbian, gay, and bisexual youth of all genders are disproportionately at risk of sexual exploitation, although the majority of sexually exploited youth still identify as heterosexual [21]. This raises an important but often overlooked element of research—the gender of the traffickers or purchasers. This is important because law enforcement requires accurate information about exploiters in order to effectively recognize and target exploiters for prevention and intervention. The majority of research identifies men as the primary exploiters of girls and young women, although in a systematic review of research about sexually exploited boys, Moynihan and colleagues identified six studies that reported both men and women sexually exploited boys, while three more studies only referred to men buying sex from boys [26]; the majority of these studies were from low-income countries such as Brazil, Ethiopia, Nigeria, Sudan, and Thailand, and most did not provide details about the prevalence of boys who were exploited by which gender. One study from Sweden reported that, among boys in their study, 48% reported sex purchased by women only, and another 8% purchased by both women and men, while 36% of boys reported sex purchased only by men, and 8% were missing the information [13]; recalculating those estimates only among those who provided a response, 74% of boys reported sex purchased by women. 

Among studies of sexual exploitation that include both boys and girls, there are very few that compare their experiences or disaggregate by gender, so it is unclear to what extent contexts for boys might be similar to or different from the experiences documented about the sexual exploitation of girls. Similarly, while homelessness or street involvement is also a risk factor for sexual exploitation, there is limited recent research in high-income countries that examines the contexts of sexual exploitation among boys who are homeless or couch-surfing; most of this research is from low-income countries [3]. In one clinical study of 62 sexually exploited adolescents seen at a Child Advocacy Centre in the United States, there were only seven boys, and insufficient power to detect statistical differences [27]. However, in the qualitative analysis of forensic interviews, they noted that the majority of boys as well as a small number of girls did not have a pimp or trafficker, directly arranging their exploitation with purchasers through the internet. Similarly, more boys than girls in the study reported being homeless, while girls were more likely to be living with at least one parent. In a school-based survey of sexual exploitation in a resort community in Canada, boys were somewhat more likely to be homeless or couch-surfing than girls, although the large majority still lived with their parents [14]; other predictors, such as binge drinking, more serious drug use, depressed mood, and impulsivity, were similar for girls and for boys. In a population-based study among Swedish adolescents in 2009, they found almost no differences in the family demographics or contexts of selling sex between boys and girls [13]. Very few studies report how many times adolescents have been exploited; in the Swedish study by Fredlund and colleagues, more than half of boys reported selling sex two to five times (52%), while girls were more likely to have reported selling sex only one time (62%) [13].

Thus, while some research with school-based or household populations and some with homeless youth samples describes the prevalence of sexual exploitation among boys, and a limited amount of community and clinical research has touched on some of the experiences of exploitation for boys, there is far less research for them than there is for girls, especially with regard to the contexts of exploitation in high-income countries such as Canada. Given this limited research, the purpose of our descriptive study was to provide information about the prevalence of and contexts within which homeless and street-involved boys report experiencing sexual exploitation in western Canada, drawing on multi-city surveys conducted in both 2006 and 2014. Furthermore, as the surveys were conducted with the same methods, eight years apart, we also compared the survey results between years, to identify whether there have been any changes in the prevalence or the contexts of sexual exploitation of homeless and street-involved boys. 

## 2. Materials and Methods

### 2.1. Surveys

This study analyzed data from the 2006 and 2014 Homeless and Street-Involved Youth (HSIY) Surveys, which were developed and administered by the McCreary Centre Society, a non-profit organization that conducts youth health research in British Columbia (BC), Canada. Data collection occurred between October 2006 and January 2007 for the first survey, and between October 2014 and January 2015 for the second survey. 

The surveys were a convenience sample of homeless and street-involved youth in multiple cities and small towns across British Columbia, the westernmost province of Canada. The youth surveyed in 2006 were from 9 different communities (n = 762) and the youth in 2014 were from 13 different communities (n = 681). Youth were recruited with the help of local agencies, and also approached in public places and on the streets where they regularly spent time. The surveys were conducted by research teams that consisted of a youth agency worker as well as a current or former street-involved youth [21]. Participants were given both written and verbal information about the research for consent, but in order to ensure the anonymity of participants from such a small and marginalized group, completion of the survey was considered consent to participate. Participants were told they could skip any question they did not wish to answer or stop the survey at any time. During survey administration, research teams read the survey aloud in case youth had literacy issues. Data collection was conducted at different times and locations in each community, to ensure most eligible youth were reached. Participants were given a CAD 10 gift card for participation in 2006, and a CAD 15 gift card in 2014. The University of British Columbia Behavioural Research Ethics Board approved the procedures for both studies.

### 2.2. Participants

In 2006, youth aged 12–18 were recruited, and the 2014 survey expanded the age range to youth aged 12–19. In their systematic review of the research about sexually exploited boys, Mitchell and colleagues [2] recommend that studies about sex exchange among adolescents and young adults, or studies of sex workers that include youth under age 18, should disaggregate those who are currently within the age definitions of sexual exploitation from those who are 18 or older, in order to provide clear inclusion criteria for systematic reviews and meta-analyses. Most studies do not include questions that ask retrospectively about age at first sex exchange, as ours does, to allow for identifying older youth who were sexually exploited, and we feel it is important to include their previous experiences. In keeping with Mitchell et al.’s recommendations, two key groups of sexually exploited boys were considered in this study: (a) boys who were between the ages of 12–17 at the time of the survey and indicated that they had been sexually exploited; and (b) boys who were age 18 or 19 at the time of the survey, and indicated that they were first sexually exploited when they were younger than 18 years of age. In this article, we use the terms older and younger boys to refer to youth 18 years of age and older and 17 years of age and younger, respectively. Table 1 describes the demographics of the sample. It should be noted that the sample in each year included self-reported cisgender males only. Fewer than 10 transgender boys participated in either survey year, so separate analyses for them could not be conducted, and previous research suggests their experiences are likely to be different enough from cisgender boys that they should not be grouped together [28]. 

### 2.3. Measures of Sexual Exploitation

Questions about sexual exploitation in the 2006 survey were drawn from a study of sexually exploited youth in 4 cities in BC conducted in 1998 [29], which had also been used in two earlier surveys of homeless and street-involved youth, one conducted in 1994 in Vancouver [30], and another in 6 large and small cities across BC in 2000 [31]. These questions were further reviewed by an advisory group of staff from more than 10 health and social service agencies who worked with street-involved youth and adolescents who were sexually exploited, to identify whether the questions needed to be adapted for more current slang terms. The surveys were also piloted with the group of youth co-researchers who had experience of homelessness and sexual exploitation, to ensure clarity and relevance. In the 2014 survey, many of the same questions were asked, which allowed for comparisons of data from both surveys. Questions in the survey that were measures of sexual exploitation did not use the term “sexual exploitation” directly, because adolescents identified that as stigmatizing, and prone to denial due to social desirability bias [21]. Instead, questions on both the 2006 and 2014 surveys used more neutral terms like “exchanging sex” or “trading sex.” In 2006, the section on sexual exploitation started with an explanatory box that said: “The next set of questions ask about engaging in sexual activities in exchange for money or goods. Sexual activities may include vaginal sexual intercourse, masturbation, fondling, sexual touching, oral sex, anal sex, posing etc. Goods may include food, cigarettes, clothing, shelter, friendship, drugs, alcohol, or transportation etc.” After this preamble, youth were asked if: (a) they had ever “engaged in any sexual activity with a male in exchange for money or goods” and the same question with a female; (b) what they had traded sex for, with options of: food, clothing, shelter (a place to stay), transportation, money, drugs or alcohol, or other items, with an open text box to add the items; (c) whether they traded sex for a “pimp, escort agency, to support a friend, partner, or relative,” with “mark all that apply” as the response options; (d) where they were living when they first traded sex, such as with family; in a group or foster home; in a shelter or safe house; in a hostel, hotel or motel; with a friend, boyfriend or girlfriend; in their own place; couch surfing; or on the street; and (e) where they exchanged sex, such as in a hotel, a club/bar, a bath house, a massage parlor, a trick pad, on the internet, or on the street (this question was only asked in the 2006 survey). Participants were also asked about their age when they first traded sex. If boys responded “yes” to any of the 6 questions in the 2006 and 5 in the 2014 surveys, they were classified as having been sexually exploited. 

Youth were also asked about a variety of other topics, such as their life experiences, physical and emotional health issues, exposure to risks, healthy behaviours and behaviours that could cause health challenges, as well as support in their lives. Many of these questions were drawn from the province-wide BC Adolescent Health Survey for youth in school, while the questions more directly related to experiences of homelessness and street involvement were taken from the prior surveys from 1994 and 2000. 

### 2.4. Analyses

Given there is little to no information about the context of sexual exploitation for boys in Canada, we primarily report descriptive analyses, performed using SPSS Version 24, including basic frequencies, cross-tabulations, valid percentages and calculations of medians and means, reported separately in younger and older groups of sexually exploited boys (age 12–17 and age 18+) in both 2006 and 2014. The first analyses were to identify the prevalence of boys who report sexual exploitation, and drew on the entire sample of boys in the survey, but the rest of the analyses were among the sexually exploited younger and older boys only. Data are reported separately by year of survey, with chi-square tests to compare the percentages between each year, within each age group. However, to investigate the experience of sexual exploitation according to the gender of perpetrators (i.e., male perpetrator only, female perpetrator only, or both male and female perpetrators), the data from both 2006 and 2014 were merged to increase the sample size in SPSS (IBM Corporation, Armonk, NY, USA). A one-way ANOVA was run to compare sexual exploitation experiences between gender of exploiters, age of boys, and age of boys when they first traded sex. 

Pair-wise deletion was used to account for missing data, because imputation in a sample from a population whose underlying denominator is unknown may introduce errors that are equally unmeasurable. Our evaluation of the dataset suggested that some of the missingness appears to be at random, and some appears to not be random; a trial imputation of the results greatly overestimated the proportion of sexually exploited boys compared to the original dataset, raising further concerns about the appropriateness of using imputation with samples from marginalized groups such as these.

## 3. Results

### 3.1. Sample Characteristics

Table 1 provides a description of all boys included in the surveys. Among all boys surveyed, 132 boys indicated they had been sexually exploited, or just over one in four younger adolescents, and one in 10 older adolescents. The majority of sexually exploited boys were aged 12–17 years, compared to boys who were 18+. The median age boys first traded sex was under 18 years of age for all boys in both surveys (see Table 1). Among younger youth, the median age they first traded sex was 14 years for both surveys; and older youth (>18+ years) stated they first traded sex at a median age of 15 (2006 survey) or at 17 years (2014 survey). A large percentage of boys who were sexually exploited identified as heterosexual or straight, followed by bisexual and gay. Among boys of 12–17 years of age in both surveys, approximately 12% stated they were unsure about their sexual orientation or did not have attractions; no boys 18+ identified uncertainty or absence of sexual attractions. There were no differences in the proportion of exploited youth by racial or ethnic minority categories within either year, although among boys aged 12–17 years, a larger proportion of exploited youth in 2014 reported having been born in Canada compared to youth in 2006 (*X*^2^ *=* 4.05, df = 1, *p* < 0.05). 

### 3.2. Genders of Exploiters

**Figure 1:**
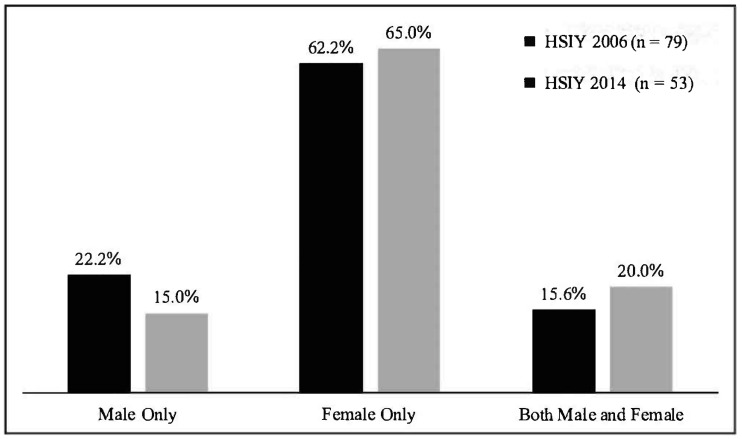
Genders of exploiters reported by sexually exploited boys.

The overwhelming majority of sexually exploited boys (77.8% and 85%) reported they were exploited by women in both the 2006 and 2014 surveys (see Figure 1). Of boys who were sexually exploited by women, 62%–65% were exploited by women only, while 15%–20% reported being exploited by *both* men and women. Men were identified as the only perpetrator by 15%–22% of boys; these percentages were not significantly different between years. With the one-way ANOVA comparing the experiences of sexual exploitation by age of sexually exploited boys, age they first traded sex, and gender of perpetrators, there was a significant effect of the age of boys who were sexually exploited and the gender of perpetrator (males only, female only and both male and female) at *p* < 0.05 for the three conditions (F (2, 62) = 3.178, *p* = 0.049). Post hoc analyses using Tukey’s Honestly Significant Difference indicated that boys who reported being sexually exploited by females only were older on average (M = 16.41) than those who said they were exploited by both male and females (M = 15.09). No differences were noted between gender of perpetrators and the age at which boys first traded sex.

### 3.3. Housing Situations

Table 2 summarizes the housing situation of all boys who were sexually exploited, and includes boys living in precarious housing as well those who have been in government care. Questions that asked about precarious housing situations included the following: living in a shelter, squat, on the street, in an abandoned building, in a tent or car, living “nowhere” or “all over,” or couch surfing.

Over half of all sexually exploited boys aged 12–17 years in both surveys had previously lived in precarious housing, while all older boys in 2014 had previously lived in precarious housing, which was significantly higher than in 2006 (*X*^2^ *=* 33.97, df = 1, *p* < 0.01). Older boys (18+ years) were much more likely to identify currently living in precarious housing, whereas fewer than one third were living in precarious housing among younger boys, but both groups were more likely to currently be living in precarious housing in 2014 compared to 2006 (both *p* < 0.01). Housing instability was revealed by the varying length of time sexually exploited boys were at their current address, and a significantly greater percentage of sexually exploited older boys in 2014 had lived at their current address for <1 month compared to older boys in the 2006 survey (*X*^2^ *=* 111.35, df = 1, *p* < 0.001). 

Among boys who were sexually exploited, the percent of boys aged 12–17 who had ever been in government care, such as a foster home, was significantly greater in the 2014 survey (*X*^2^ *=* 44.08, df = 1, *p* < 0.001); there were no significant differences between survey years in younger boys who had ever been in group homes. Although the percent of boys 18+ who had been in foster care was larger in 2014 compared to 2006 (*X*^2^ *=* 12.84, df = 1, *p* < 0.01), the percent of boys 18+ in group homes was significantly lower in 2014 than in 2006 (*X*^2^ *=* 34.17, df = 1, *p* < 0.001). The percentage of boys who had ever been in a custody center was higher in 2006 than in 2014 among all sexually exploited boys (both *p* < 0.001). While no youth agreements were available in 2006 (youth agreements are a living arrangement that allows youth to live independently and is an alternative to government care [21]), the 2014 survey reported just under 30% of boys lived in this alternative arrangement.

### 3.4. Contexts and Venues of Sexual Exploitation

Table 3 provides details about how sex was exchanged in sexual exploitation. The most common reason in 2014 boys traded sex was to support a friend, partner or relative, and the second most common was trading sex for a pimp (different from 2006, *p* < 0.01). Over half of boys sexually exploited in 2014 traded sex to support a friend, partner or relative. In contrast, in 2006, youth most commonly indicated neither pimp, escort agency or friend, partner or relative, with the second most common being friends or relatives (both significantly different from 2014, both *p* < 0.001). The most common types of consideration sexually exploited boys received for sex were money, drugs or alcohol, or shelter, and food, clothing, and transportation were the least common, but none of these differed significantly between 2006 and 2014. A large number of boys reported living with their family when they were first exploited (25.5% of boys in 2006 and 41.2% in 2014, although not significantly different). “On the street” was another common place where boys were living when first exploited, and this proportion was considerably higher in 2014 (35.3%) compared to 2006 (19.1%, *X*^2^ *=* 4.37, df = 1, *p* < 0.05), as was couch surfing and living in their own place (both 17.6% vs. 6.4%, *X*^2^ *=* 4.10, df = 1, *p* < 0.05). Alternately, 8.5% of boys in 2006 were living in foster care when they were first exploited, but no boys in 2014 reported living in foster care when they first were exploited (*p* < 0.05).

### 3.5. Risks and Consequences Associated with Sexual Exploitation among Boys

#### 3.5.1. Which Came First? Timing of Exploitation vs. Homelessness

Table 4 compares whether homelessness, running away or being kicked out occurred before or after the age boys reported they were first exploited. Among all sexually exploited boys who were surveyed, over half of the boys reported being homeless before being exploited. In 2006, 22.9% of boys indicated that they traded sex and became homeless at the same age, as did 20% in 2014; however, in 2014, the same percent of boys stated they had traded sex first as the number who said they traded sex and became homeless at the same age; these results were significantly different between years (*X*^2^ *=* 8.41, df = 2, *p* < 0.05). Nearly half (45.7%) of the exploited boys in 2006 reported running away from home before being exploited, and this number increased to greater than half (55.6%) among boys in 2014 (*X*^2^ *=* 6.46, df = 2, *p* < 0.05). Although fewer boys reported trading sex before running away, this number remained similar in both surveys. Boys who ran away from home the same year they first traded sex was lower among boys who were surveyed in 2014. Boys who were kicked out of their home first or traded sex first were similar in 2006; however, this number was higher in 2014 and the discrepancy between the two also widened (*X*^2^ *=* 9.80, df = 2, *p* < 0.01). Similar to boys who ran away from home and first traded sex during the same year, the percent of boys who were kicked out and first traded sex within the same year was lower in 2014.

#### 3.5.2. History of Sexual Abuse

Although sexual exploitation is a form of sexual abuse and therefore 100% of sexually exploited boys could have reported being sexually abused, fewer than one in three sexually exploited boys in each year reported they had been sexually abused (29.0% in 2006, 27.5% in 2014, not significantly different). There was a slight change in one question about sexual abuse (forced sex) between the 2006 and 2014 survey (forced to have sexual intercourse vs. forced to have sex, see Table 5—note that this is not specifically forced to have sex in exchange for money or consideration, it is a more general question about being forced by someone to have sex). Among boys who were sexually exploited, they more often reported being forced to have sex by another youth rather than by an adult; compared to boys in 2006, boys in 2014 were significantly more likely to report either not being forced to have sex by anyone or being forced to have sex by another youth (*p* < 0.01 and *p* < 0.05, respectively). Table 5 also lists the specific people they reported had sexually abused them. A similar number of boys indicated they had been sexually abused by family members and non-family members. The percent of boys sexually abused by various people was similar in 2006 and 2014, and even apparent differences were not statistically significant. Only a small number of boys reported being sexually abused by a trick or date, a pimp or agency manager and by a police officer.

## 4. Discussion

This study reported the prevalence and experiences of sexual exploitation among adolescent boys aged 12–19 in multiple cities in two surveys of homeless and street-involved adolescents in western Canada. Just over one in four boys ages 17 and under reported trading sex for money or other consideration, which in Canada is a form of sexual exploitation, and which is within the mid-range among estimates that other surveys of homeless youth have reported [18,19,20,21,22,23], but far higher than school population surveys report [12,13,14,15,16,17]. A further 1 in 10 youth who were 18 years or older reported first trading sex at age 17 or younger, which also fits the legal definition of sexual exploitation in Canada. The majority of sexually exploited boys ran away, were kicked out, or became homeless before first being sexually exploited, although about a third reported first trading sex while living with their family, which raises questions about whether their families were the ones trafficking them, or if they were being exploited without their family’s knowledge. We were unable to determine this from the survey questions. As none of the existing limited research about sexually exploited homeless boys included information on whether they were first exploited before or after becoming homeless other than our earlier exploration of the 2006 survey data [8], and the population-based studies only identified a higher risk of running away or homelessness predicting sexual exploitation but not actually calculating which came first, we identify novel findings of two primary patterns of exploitation: one while living within the family, and the other while on the street. The majority had also been in government care at some point in their lives, underscoring another key vulnerability, where young people were disconnected from family and may be less protected, or may need to exchange sex as a means for survival. 

Both adolescents under the age of 18 and those 18 and older reported sexual exploitation first occurring as young as age 10 or 11, with a median age among younger adolescents of exploitation at 14 in 2006 and in 2014, and at slightly older ages among 18- and 19-year-olds. These age ranges are similar to those in most other studies. 

A relatively small percentage of youth reported exchanging sex for a pimp or trafficker, or an escort agency, instead reporting they were trading sex to support a friend, partner, relative, or trading sex on their own. This is similar to the findings in the US in one study, where the majority of boys (although a small group) were exploited without a pimp, while a smaller number of girls did not have a trafficker [27]. On the other hand, in that study, many of the sexually exploited youth did not identify their trafficker as such, but rather considered them a romantic partner, or friend, identifying a significant emotional connection to their trafficker. We similarly found that the majority of boys in 2014 reported exchanging sex for a friend, partner or relative instead of for a pimp or escort agency, while in 2006, the majority of boys who had been exploited indicated trading sex for “none of the above.” It is possible that some boys interacted directly with a purchaser, without a trafficker or procurer contacting purchasers and making arrangements, as described in the study above [27]. Alternately, some boys who are being trafficked may not recognize the person as a trafficker if they are emotionally manipulated into sexual exploitation, rather than through violence or coercion.

An unexpected finding was that the large majority of boys were sexually exploited by women. The great majority of boys—77.8% or 85%-reported being exploited by women, and nearly two in three reported being exploited only by women. This is quite different from the common stereotypes of men as traffickers and purchasers, and is an even larger proportion than the estimated 74% described in the one study from Sweden that calculated the gender of purchasers [13]. The survey identified the prevalence of boys who had at least one woman exploiter but did not ask how many women boys had been exploited by. The study by Fredlund and colleagues (2013) from Sweden noted that about half of boys had sold sex two–five times, but this was a school-based study. Therefore, it is difficult to estimate the actual numbers of women who might be exploiting homeless adolescent boys. The literature also offers almost no descriptions of women who purchase sex from adolescents or adults, other than a recent study of sex tourism in the Caribbean [32]. Neither was our survey able to explore in greater detail who the women are who are purchasing sex from adolescent boys, or what they are exchanging for this sex, whether this is money, drugs, food, shelter, or other goods. More research is needed to understand the types of women who sexually exploit boys, in order for law enforcement to recognize to intervene to prevent this exploitation. Given the paucity of information, qualitative designs are a warranted first step.

A related difference in our findings was that the large majority of boys in both surveys identified as heterosexual, which differs from studies that identify a primary risk for sexual exploitation is a gay or bisexual sexual orientation [25] and the statements that assume most or all purchasers of sex from boys are men, even where the studies did not actually ask about the gender of sexual partners. While sexually exploited youth often do not have a choice about who they exchange sex with, and heterosexual boys may well trade sex for money or goods with gay men, sometimes termed “gay for pay,” our findings did not suggest this was the predominant experience of the sexually exploited boys in our study. It is unclear why our findings would differ so much from studies in other countries, but given similar results 8 years apart, it suggests stability in these findings in western Canada at the least. 

Another concern is that fewer than a third of sexually exploited boys indicated they had been sexually abused, although sexual exploitation is a form of sexual abuse, so all of them could have indicated a history of sexual abuse. This suggests that homeless and street-involved boys in Canada may not have the information to recognize exploitation as a form of sexual violence and a crime against them. Given that the pervasive discourse about sexual exploitation and sex trafficking focuses on women and girls, it is possible that boys who are exploited do not see their experiences in similar ways. Alternately, they may experience greater shame and stigma in exploitation, as it does not align with societal expectations of masculinity, and this might lead them to re-cast their circumstances in order to avoid that stigma [28]. The limited research about the health of sexually exploited boys, however, suggests they experience the same health sequelae as girls [3,27]. Interventions to help youth exit sexually exploitive relationships and circumstances must keep in mind this difficulty in identifying that exploitation is occurring, as well as the potential that youth may not want help exiting exploitation, especially if it provides a more consistent way to meet basic survival needs for food and shelter than other sources of income for homeless youth with limited education or work training. Education programs that try to prevent sexual abuse among children and adolescents should clearly include information about sexual exploitation as a form of sexual abuse or sexual violence.

Moreover, the cut-off age to define sexual exploitation as opposed to sex work is a relatively arbitrary one, given the varied timing of adolescent development for different young people; some youth may feel they have the agency and capacity to consent to sex work, while others of the same age, or older, may still be trafficked or sexually exploited, especially if fraud, force, or coercion is involved. The health consequences of sex work or sex trafficking may be similar, or the level of agency and decisional autonomy of young people involved may alter the sequelae. We were not able to determine this within our survey, which had only a few questions as part of a larger general health survey. Conversations about sexual exploitation and the sex trafficking of boys and young men need to consider their perspectives, as well as be aware of their health needs. 

There are very few evaluated interventions to help sexually exploited and trafficked youth in general with their health and psychosocial needs [26], and even fewer of these include boys, or are focused solely on boys. This may be partly because of the lack of knowledge about their experiences and the contexts they have in common with girls and transgender youth vs. contexts that are unique to boys limit the ability to develop effective, gender sensitive interventions for supporting sexually exploited boys. More research is needed to understand what works to help them, especially evaluations of interventions that either include boys, or are focused primarily on boys. 

This research has strengths and limitations that should be noted in considering the evidence it provides. First, research with homeless and street-involved populations holds unique challenges for sampling, due to the uncertainty about the true underlying population. Without an accurate denominator for the target sample, it is hard to estimate the extent to which the sample is representative, so prevalence estimates should be treated with caution. At the same time, this is a limitation of all research with homeless youth, and by sampling from multiple cities across BC, eight years apart, with experiential youth as co-researchers, we enhance the likelihood of capturing a more diverse and representative group of youth. Likewise, because our surveys were separated by eight years, we can be confident our samples are independent. Our combined sample of 132 sexually exploited boys and young men from multiples cities across Western Canada is one of the larger samples in the extant literature, even from large-scale population surveys, and as such, provides a strength in its ability to capture a wider range of experiences than smaller, more focused studies would do. However, a significant limitation that must be considered is that the questions about sexual exploitation are sensitive, even in anonymous surveys. While we tried to create non-judgmental and concrete questions for youth, we did hear objections from some participants that this information was too personal or stigmatizing, and they skipped those questions. Thus, our estimates, while in line with other studies, must be considered likely undercounts of the true extent of sexual exploitation and trafficking among homeless adolescent boys in Canada. That our attempt at multiple imputation with the full dataset resulted in a much higher number of young people identified as likely to have been sexually exploited suggests this could well be an undercount, but unfortunately, there is no way to be sure. As this study was limited to self-reported cisgender boys and young men, these analyses cannot be generalized to transgender or non-binary young people. Further research about the experiences of sexual exploitation among homeless and street-involved transgender and non-binary youth in Canada is warranted. 

Despite these strengths and limitations, it is clear that a significant proportion of adolescent homeless and street-involved boys in western Canada are experiencing sexual exploitation, some as young as 10 years old, and with a number of them exploited while living with family members. The majority of them experience homelessness or precarious housing as a precursor to their exploitation. Additionally, contrary to the general perception, women do sexually exploit boys—in the case of boys in western Canada, the large majority of those who participated in our study had been exploited by women, and a majority had been exploited solely by women. Our commitment as a society to the UN Convention on the Rights of the Child, to protect young people from trafficking and sexual exploitation, must include young people of all genders. This study helps raise our awareness of the experiences of homeless and street-involved boys and young men who have been sexually exploited as adolescents, and points to some next directions for action. 

## 5. Conclusions

Contrary to general perceptions, more than one in four homeless adolescent boys surveyed in western Canada reported sexual exploitation, and their experiences do not match stereotypes, especially the genders of their exploiters. Gender-specific interventions to better support sexually exploited and sex trafficked boys are needed to effectively address their circumstances. By disaggregating our findings by younger boys (age 17 and under), and older boys and young men (18 years and older) in adherence to international definitions of sexual exploitation, future systematic reviews or meta-analyses can incorporate results from this study, allowing global comparisons to include data from Canada. 

## Figures and Tables

**Table 1:** Description of sample.

Sample Demographics	HSIY 2006 (N = 362)		HSIY 2014 (N = 318)	
	12–17 Years (n = 238)	18+ Years * (n = 124)	12–17 Years (n = 175)	18+ Years (n = 143)
% of Sexually Exploited Males	27.3 (n = 65)	11.3 (n = 14)	26.3 (n = 46)	4.9 (n = 7)
Sexual Orientation				
Straight	68.3	75.0	73.2	71.4
Bisexual	11.7	16.7	12.2	14.3
Gay	8.3	8.3	2.4	14.3
Not sure/Don’t have attractions	11.7	0.0	12.2	0.0
Born in Canada	87.5	92.9	93.5	85.7
	Median (Range)	Median (Range)	Median (Range)	Median (Range)
Age First Traded Sex	14 (11, 17)	15 (12, 17)	14 (10, 17)	17 (13, 17)

* HSIY 2006 sampled youth 18 and under; HSIY 2014 sampled youth 19 and under.

**Table 2:** Participant housing situation of sexually exploited boys.

Housing Experiences	HSIY 2006 (n = 79)		HSIY 2014 (n = 53)	
	12–17 Years	18+ Years *	12–17 Years	18+ Years *
% Ever Lived in Precarious Housing	54.1	78.6	58.5	100.0
% Currently in Precarious Housing	15.7	71.4	29.3	85.7
Length of Time at Current Address				
<1 month	25.0	21.4	33.3	85.7
2–6 months	21.9	21.4	15.6	0.0
7–12 months	12.5	7.1	11.1	14.3
>1 year	32.8	28.6	40.0	0.0
No current address ^‡^	7.8	21.4	--	--
Ever in Government Care				
Foster home	27.9	50.0	60.5	71.4
Group home	27.9	64.3	31.6	28.6
Youth agreement *^§^*	--	--	13.5	28.6
Custody centre	55.4	84.6	17.6	33.3

* HSIY 2006 sampled youth 18 and under; HSIY 2014 sampled youth 19 and under; ^‡^ response option not provided in the 2014 HSIY survey; ^§^ response option not provided in the 2006 HSIY survey.

**Table 3:** Description of how sex was traded.

Contexts of Sexual Exploitation	HSIY 2006 (n = 79)	HSIY 2014 (n = 53)
	%	%
For Whom Sexual Activity Was Traded		
Pimp	17.9	10.4
Escort agency	4.5	8.3
Supporting a friend, partner, or relative	19.4	66.7
Other	7.5	4.2
None of the above	53.7	22.9
Respondent Traded Sex for *		
Food	5.9	14.9
Clothing	10.0	13.0
Shelter	17.3	18.7
Transportation	13.7	8.9
Money	32.4	17.1
Drugs or alcohol	29.7	18.7
Where Respondent Was Living When First Traded Sex		
With their family	25.5	41.2
In a foster home	8.5	0.0
In a group home	4.3	0.0
In a shelter or safe house	10.6	17.6
Hostel, hotel, or motel	4.3	11.8
With a friend, boyfriend, or girlfriend	10.6	11.8
In my own place	6.4	17.6
On the street	19.1	35.3
Couch surfing	6.4	17.6

* in the past 12 months.

**Table 4:** Timing of sexual exploitation vs. running away or homelessness.

Which Came First	HSIY 2006 (n = 79)	HSIY 2014 (n = 53)
	%	%
Homeless vs. Sexual Exploitation First ^†^		
Homeless first	72.9	60.0
Traded sex first	4.2	20.0
Traded sex and became homeless at same age	22.9	20.0
Running Away vs. Sexual Exploitation First ^†^		
Ran away from home first	45.7	55.6
Traded sex first	32.6	38.9
Traded sex and ran away at same age	21.7	5.6
Kicked Out vs. Sexual Exploitation First ^†^		
Kicked out of home first	37.8	52.5
Traded sex first	35.6	42.1
Traded sex and kicked out of home at same age	26.6	5.4

^†^ respondents only included if they provided an age for both survey questions.

**Table 5:** Sexual abuse reported by sexually exploited boys.

Experiences of Sexual Abuse	HSIY 2006 (n = 79)	HSIY 2014 (n = 53)
	%	%
Forced to Have: “Sexual Intercourse’ (2006) or “Sex” (2014)		
No	57.0	80.8
By another youth	26.9	11.5
By an adult	19.2	9.6
Ever Been Sexually Abused	29.0	27.5
Abused by a family member	16.9	17.8
Abused by a non-family member	19.7	20.0
By their mother	2.8	6.7
By their father	2.8	6.7
By their step-parent	4.2	2.2
By their foster parent	7.0	2.2
By another relative	9.9	4.4
By a friend	8.5	8.9
By a romantic partner	4.2	2.2
By a trick or date	5.6	4.4
By a pimp or agency manager	2.8	2.2
By a police officer	2.8	4.4
By a stranger	4.2	11.1

## Data Availability

The data sources for this study are not able to be made publicly available, due to the original research ethics protocol and participant agreements that the McCreary Centre Society implemented when conducting their surveys. Requests to access the BC Homeless and Street-Involved Youth (HSIY) Surveys by scholars must be made directly to the Executive Director of McCreary Centre Society, please see http://www.mcs.bc.ca for further details and contact information.
